# Cost-Minimization and Procedural Outcomes of Cold Snare Versus Cold Forceps Polypectomy for Small Colorectal Polyps: A Prospective Cohort Study Using Real Institutional Pricing

**DOI:** 10.3390/healthcare14162656

**Published:** 2026-08-21

**Authors:** Güney Özkaya, İsmail Ege Subaşı, Sangar Abdullah, Sertaç Doğan, Şiva Nafez

**Affiliations:** 1 Department of General Surgery, Sancaktepe Şehit Prof. Dr. İlhan Varank Training and Research Hospital, Istanbul 34785, Turkey; drsangarabdullah@gmail.com (S.A.); sertacdogan34@gmail.com (S.D.); 2 Department of Gastroenterology Surgery, Sancaktepe Şehit Prof. Dr. İlhan Varank Training and Research Hospital, Istanbul 34785, Turkey; dregesubas@gmail.com; 3 Department of Pathology, Sancaktepe Şehit Prof. Dr. İlhan Varank Training and Research Hospital, Istanbul 34785, Turkey; shiva.nafez@gmail.com

**Keywords:** colonoscopy, colorectal polyps, cold snare polypectomy, cold forceps polypectomy, cost savings, health care costs, value-based care, real-world evidence

## Abstract

**Background/Objectives:** Although current guidelines (European Society of Gastrointestinal Endoscopy [ESGE] 2024, U.S. Multi-Society Task Force [USMSTF] 2020) recommend cold snare polypectomy (CSP) over cold forceps polypectomy (CFP) for small colorectal polyps, CFP remains widely used. Comparative economic data using real institutional pricing are scarce, limiting evidence for guideline-concordant resource allocation. We evaluated the healthcare economics and procedural outcomes of CSP versus CFP to assess their institutional resource implications. **Methods:** This prospective cohort study analyzed 153 consecutive patients (181 polyps ≤ 9 mm) at a single tertiary care center (June–October 2025). Technique allocation was based on endoscopist preference. The primary outcome was histopathologically confirmed complete resection; secondary outcomes were one-piece resection rate, procedure time, and cost outcomes based on institutional pricing. Firth penalized logistic regression, propensity score matching, and a neoplastic-restricted sensitivity analysis were performed. **Results:** Complete resection rates did not differ significantly (CSP 97.4% vs. CFP 94.2%, *p* = 0.29). CSP achieved higher one-piece resection rates (96.2% vs. 84.5%, *p* = 0.011) and shorter procedure time (median 3.2 vs. 5.1 min, *p* < 0.001). For 6–9 mm polyps, piecemeal resection was reduced with CSP (6.8% vs. 28.6%, *p* = 0.010). Despite higher device costs ($6 vs. $3), direct device-plus-labor cost was lower for CSP ($18.06 vs. $22.24, *p* < 0.001), with no statistically significant difference in complete resection. High-grade dysplasia was the only variable independently associated with incomplete resection. Findings were consistent in propensity score-matched (*n* = 61 pairs) and neoplastic-restricted (*n* = 134) analyses. **Conclusions:** By reducing direct device-plus-labor costs by 19% while improving one-piece resection, and remaining consistent across multivariable, propensity score-matched, and sensitivity analyses, these findings indicate that, within a cost-minimization framework, CSP is a cost-saving, guideline-concordant technique with no statistically significant difference in complete resection or observed adverse events.

## 1. Introduction

Colorectal cancer (CRC) is the third most common cancer worldwide and the second leading cause of cancer-related mortality, with most cases arising from adenomatous polyps through the adenoma–carcinoma sequence [1]. Colonoscopic polypectomy prevents colorectal cancer and reduces mortality [2]. Because incomplete resection markedly increases the risk of interval cancers and post-colonoscopy CRC [3,4], complete polyp removal is essential; 10–27% of interval CRCs may be attributable to incomplete polyp resection, underscoring the importance of achieving complete histologic eradication [5].

Small (6–9 mm) and diminutive (≤5 mm) colorectal polyps represent the majority of lesions detected during screening colonoscopy [6]. For these lesions, guidelines from both the U.S. Multi-Society Task Force (USMSTF) and the European Society of Gastrointestinal Endoscopy (ESGE) recommend cold snare polypectomy—performed without electrocautery—given its favorable safety profile, with lower risks of delayed bleeding, perforation, and post-polypectomy syndrome [7,8,9].

Two primary cold polypectomy techniques are available: cold snare polypectomy (CSP) and cold forceps polypectomy (CFP). Multiple randomized controlled trials have compared these techniques, demonstrating superior one-piece resection rates with CSP [10,11]. Accordingly, the 2020 USMSTF guidelines strongly recommend CSP and recommend against CFP for diminutive and small polyps because of high rates of incomplete resection [7], and the updated 2024 ESGE guidelines likewise strongly recommend CSP while recommending against CFP for these lesions [8].

Because both CSP and CFP are performed without electrocautery, they share a comparable safety profile; the choice between them therefore turns on resection quality and economic considerations rather than safety—yet the economic dimension remains poorly characterized. Despite guideline recommendations for CSP, CFP remains widely used in practice, and the economic implications of choosing CSP over CFP are not well understood. Although CSP devices have higher acquisition costs than biopsy forceps, the overall cost implications—accounting for procedure time and labor costs—have not been well characterized. Given the high volume of screening colonoscopy, even small per-procedure differences in cost and time may aggregate into meaningful resource implications at the health-system level. While the cost-effectiveness of endoscopic mucosal resection versus surgery for large laterally spreading lesions has been established using institutional cost data, comparative cost analyses between CSP and CFP for small polyps (≤9 mm) using real institutional device pricing and measured labor costs are lacking [12], restricting evidence-based resource allocation decisions.

Therefore, this study aimed to evaluate the healthcare economics and procedural outcomes of CSP versus CFP for small colorectal polyps (≤9 mm) using real institutional device pricing and measured procedure times to calculate actual labor costs. We hypothesized that the time efficiency of CSP might offset its higher device costs, resulting in comparable or lower direct device-plus-labor costs.

## 2. Materials and Methods

### 2.1. Study Design and Setting

This prospective, non-randomized observational cohort study was conducted at a tertiary care teaching and research hospital. Polypectomy technique (cold snare or cold forceps) was selected at the discretion of the performing endoscopist rather than by randomization. The study included consecutive eligible patients who underwent colonoscopy with polypectomy for small colorectal polyps between June 2025 and October 2025. The study protocol was approved by the institutional ethics committee (approval number: 2025/201, date: 4 June 2025) and was conducted in accordance with the Declaration of Helsinki. Written informed consent was obtained from all patients prior to inclusion. Screening logs were not systematically maintained. All eligible patients identified during routine clinical practice were enrolled consecutively.

### 2.2. Patient Selection

Inclusion criteria were: (1) age ≥ 18 years; (2) presence of at least one colorectal polyp ≤9 mm detected during screening, surveillance, or diagnostic colonoscopy; and (3) polyp amenable to cold snare or cold forceps polypectomy.

Exclusion criteria were: (1) inadequate bowel preparation (Boston Bowel Preparation Scale (BBPS) score < 6); (2) inflammatory bowel disease (Crohn’s disease or ulcerative colitis); (3) familial adenomatous polyposis or other hereditary polyposis syndromes; (4) prior colorectal surgery affecting the polyp site; (5) therapeutic anticoagulation requiring heparin bridging or high bleeding risk; (6) pregnancy; (7) polyps requiring hot snare polypectomy or endoscopic mucosal resection based on size or morphology.

### 2.3. Polypectomy Techniques

All procedures were performed by four endoscopists, each experienced in both cold snare and cold forceps techniques and each performing approximately 1000 colonoscopies annually. High-definition colonoscopes were used for all procedures.

Polypectomy technique selection was based on endoscopist judgment considering polyp size, morphology, location, and anticipated technical difficulty. This approach reflects real-world clinical practice patterns at our institution. Polyp size was estimated using open biopsy forceps (approximate span 7 mm) as reference. Polyp location was classified as proximal (cecum to splenic flexure) or distal (descending colon to rectum). Polyp morphology was classified according to the Paris classification (0-Is, 0-IIa, 0-Ip, 0-Isp, 0-IIb) by the performing endoscopist [13].

Cold snare polypectomy was performed using single-use, CE-marked thin-wire cold polypectomy snares (10–13 mm diameter). The polyp was encircled at its base, ensuring capture of 1–2 mm of surrounding normal mucosa. The snare was then closed firmly and the polyp was transected mechanically without application of electrocautery. Retrieved specimens were sent for histopathologic examination. Base biopsies (two samples) were obtained from the mucosa at the center of the resection base, without separate sampling of the lateral margins of the post-polypectomy defect, using standard biopsy forceps to assess completeness of resection. Histopathologic specimens were evaluated by a gastrointestinal pathologist as part of routine clinical reporting, with difficult or equivocal cases reviewed in consensus. Pathologists were not blinded to the polypectomy technique, as this information was available from the colonoscopy reports. Cold forceps polypectomy was performed using single-use, CE-marked standard-capacity (non-jumbo) cold biopsy forceps (2.4 mm cup diameter). The polyp was grasped with the forceps and removed mechanically without electrocautery. For larger polyps within the diminutive and small size range, multiple bites were performed until the polyp appeared completely removed. Base biopsies (two samples) were similarly obtained from the mucosa at the center of the resection base, without separate sampling of the lateral margins of the post-polypectomy defect, using standard biopsy forceps to assess completeness of resection. Both devices were procured through the centralized public tender of the State Supply Office (Devlet Malzeme Ofisi, DMO, Istanbul, Turkey); individual device models were not recorded and are therefore not specified.

### 2.4. Outcome Measures

The primary outcome was complete resection rate, defined as histologically confirmed absence of residual lesional tissue (defined as polyp tissue corresponding to the histologic type of the index polyp) in the sampled base biopsies. Secondary outcomes included: (1) one-piece resection rate, defined as en bloc removal without piecemeal technique (single application of snare or forceps removing the entire polyp); (2) procedure time, measured from first application of the polypectomy instrument to polyp retrieval; (3) piecemeal resection rate (removal requiring ≥2 applications); (4) direct device-plus-labor cost; and (5) procedural cost outcomes, analyzed within a cost-minimization framework (direct device-plus-labor cost and cost per complete resection), with procedural outcomes presented alongside costs (cost-consequence presentation). Histopathologic evaluation was performed according to the World Health Organization classification. Advanced adenoma was defined as an adenoma with villous histology, high-grade dysplasia, or size ≥ 10 mm. Procedure-related complications were systematically recorded at the patient level, including perforation, delayed post-procedural bleeding (defined as clinically significant bleeding occurring within 30 days after polypectomy), and intraprocedural (immediate) bleeding requiring endoscopic intervention.

### 2.5. Cost Analysis

Cost analysis was conducted from the institutional (health-care provider) perspective and included direct material and labor costs. Material costs were obtained from the hospital purchasing department and reflect the actual per-unit acquisition prices awarded under the DMO lowest-price public tender, in which contracts are granted to the lowest-cost compliant product. Costs were originally incurred in Turkish lira (TRY) under 2025 institutional contracts and converted to U.S. dollars (USD) using the mean TRY/USD exchange rate over the study period (June–October 2025; 1 USD ≈ 40.75 TRY), sourced from the Central Bank of the Republic of Türkiye (CBRT). Device costs were the average awarded unit prices of $6.00 for cold snares and $3.00 for cold biopsy forceps. Labor costs were derived from institutional payroll and overhead accounting, yielding an approximate blended rate of $3 per minute ($180 per hour) for endoscopy personnel (nurses and physicians), inclusive of direct salary and allocated overhead. Direct device-plus-labor cost was calculated as material cost plus labor cost (procedure time × labor rate per minute). Cost per complete resection was calculated as direct device-plus-labor cost divided by the complete resection rate for each technique. The ICER was calculated as the difference in mean direct device-plus-labor cost divided by the difference in complete resection rates. Because the primary economic comparison was framed as a cost-minimization analysis under an assumption of comparable effectiveness, cost per complete resection and the ICER are reported as secondary descriptive measures rather than as evidence of cost-effectiveness. Consistent with this provider-perspective scope, the analysis was limited to direct device and procedural labor costs (the latter inclusive of allocated personnel overhead); sedation, pathology processing, repeat procedures, and complication management were not included.

### 2.6. Statistical Analysis

No a priori sample-size calculation was performed, and no minimum clinically important difference was pre-specified; the study included all consecutive eligible patients during the enrollment period and was therefore not formally powered for the complete resection endpoint. Continuous variables were tested for normality using the Shapiro-Wilk test (Table S1). Non-normally distributed data are presented as median (interquartile range (IQR)) and compared using the Mann-Whitney U test. Normally distributed data are presented as mean ± standard deviation. Categorical variables are presented as number (percentage) and compared using the χ^2^ test or Fisher’s exact test as appropriate. Unadjusted odds ratios (OR) and risk differences (RD) with 95% confidence intervals were calculated for binary outcomes using logistic regression and linear risk models, respectively. In addition to inferential testing, standardized mean differences (SMDs) were calculated for all baseline characteristics in Table 1 and Table 2 to quantify effect-size magnitude of between-group imbalance independent of sample size [14]. An |SMD| > 0.10 was used to indicate meaningful imbalance [14], with |SMD| > 0.25 considered a large imbalance [15]. For continuous variables, SMDs were calculated from means and standard deviations even when medians and IQRs are reported. For multi-level categorical variables (ASA, Paris classification, histopathology), SMDs were calculated for each category separately. Exploratory subgroup analyses by polyp size category (≤5 mm vs. 6–9 mm) were performed for the primary and secondary outcomes. As 28 patients contributed two polyps, the influence of intra-patient clustering on the binary outcomes was assessed using cluster-robust (Huber–White) standard errors with the patient as the clustering unit, and in a sensitivity analysis restricted to one randomly selected polyp per patient (Tables S2 and S3). The robustness of the primary outcome to the two CSP polyps not retrieved for histopathology was examined in a worst-case analysis in which these were reclassified as incomplete resections (Table S4). The robustness of the primary outcome to potential outcome misclassification, arising from the lack of pathologist blinding, was further assessed by reclassifying a plausible range of complete resections as incomplete under both non-differential and technique-differential assumptions (Table S5). The sensitivity of the observed associations to unmeasured confounding was quantified using *E*-values [16], calculated on the risk-ratio scale for the primary and key secondary outcomes (Table S6).

Given the low number of incomplete resection events (*n* = 8, 4.4%; approximately 2 events per covariate), Firth penalized maximum likelihood logistic regression was performed to identify independent predictors of incomplete resection, addressing potential small-sample bias in conventional logistic regression [17]. The model included four covariates: polypectomy technique (CSP vs. CFP), polyp size (6–9 mm vs. ≤5 mm), polyp location (proximal vs. distal), and high-grade dysplasia (present vs. absent). 95% confidence intervals and *p* values were computed using profile likelihood, as recommended for Firth-type models. Results are presented as odds ratios (OR) with 95% CIs.

For the cost-minimization analysis, 95% confidence intervals for cost differences and ICER were estimated using bootstrap resampling with 10,000 iterations. Statistical analyses were performed using IBM SPSS Statistics version 26.0 (IBM Corp, Armonk, NY, USA). Firth penalized logistic regression was performed using the STATS FIRTHLOG extension for SPSS. Two-sided *p* values < 0.05 were considered to indicate statistical significance. Sensitivity analysis was performed to assess the robustness of cost-minimization findings across different labor cost assumptions. Direct device-plus-labor costs were recalculated using labor rates of $2, $3, and $4 per minute, and the statistical significance of cost differences was assessed at each rate. In addition, a break-even labor rate was derived analytically as the rate at which the higher device cost of CSP would be exactly offset by its shorter procedure time, below which the cost advantage would no longer hold.

To address potential confounding from non-randomized technique selection, propensity score matching was performed as a sensitivity analysis. A propensity score representing the conditional probability of receiving CSP was estimated using logistic regression with eight covariates: polyp size (continuous), multiple lesion status (binary), polyp location (proximal vs. distal, binary), BBPS (continuous), and Paris classification (four dummy variables for 0-Is, 0-IIa, 0-Ip, 0-Isp, with 0-IIb as reference). Demographic variables (age, sex, BMI) were not included as matching covariates because the matching unit was the polyp rather than the patient. High-grade dysplasia was not included as a matching covariate, as histopathology was determined after polypectomy and was therefore not available at the time of technique selection. One-to-one nearest-neighbor matching without replacement was performed using a caliper width of 0.2× the standard deviation of the logit of the propensity score, as recommended by Austin [18]. Matching was performed with a fixed random seed to ensure reproducibility. Balance in the matched cohort was assessed using SMDs as defined above (Figure S1); |SMD| < 0.25 was considered to indicate acceptable balance, per the same methodological recommendations [15]. The *p* values were not used to assess balance in the matched cohort, in line with current methodological recommendations for propensity score matched analyses [19].

Outcomes in the matched cohort were analyzed using paired statistical methods that account for the matched-pair structure. McNemar’s exact test was used for paired binary outcomes (complete resection, one-piece resection, piecemeal resection), and the Wilcoxon signed-rank test was used for paired continuous outcomes (procedure time, direct device-plus-labor cost). In the matched cohort, risk differences (RD) with 95% confidence intervals estimated by bootstrap resampling (10,000 iterations at the matched-pair level) are reported as the primary effect measure for each binary outcome in the main text, as RDs provide clinically interpretable absolute effect sizes. Paired odds ratios, calculated as the ratio of discordant pairs (CSP-favoring/CFP-favoring) with 95% CIs by the Wald log-OR method and Haldane–Anscombe continuity correction (+0.5 added to each cell) when zero discordant pairs were observed in one direction, are reported in Table 3 alongside their full discordant pair counts. An exploratory subgroup analysis restricted to matched pairs in which both members had polyps of 6–9 mm was performed. Propensity score matching was implemented using the STATS PSMATCHING extension for SPSS.

Given that the primary outcome is most directly assessed in neoplastic lesions where residual dysplastic tissue can be clearly identified, a sensitivity analysis was performed restricted to polyps with low-grade or high-grade dysplasia (*n* = 134; 58 in the CSP group and 76 in the CFP group), excluding non-neoplastic lesions (*n* = 47). The same statistical approaches as for the full cohort were applied to all primary and secondary outcomes; detailed results are presented in Table S7.

## 3. Results

### 3.1. Patient and Polyp Characteristics

**Figure 1:**
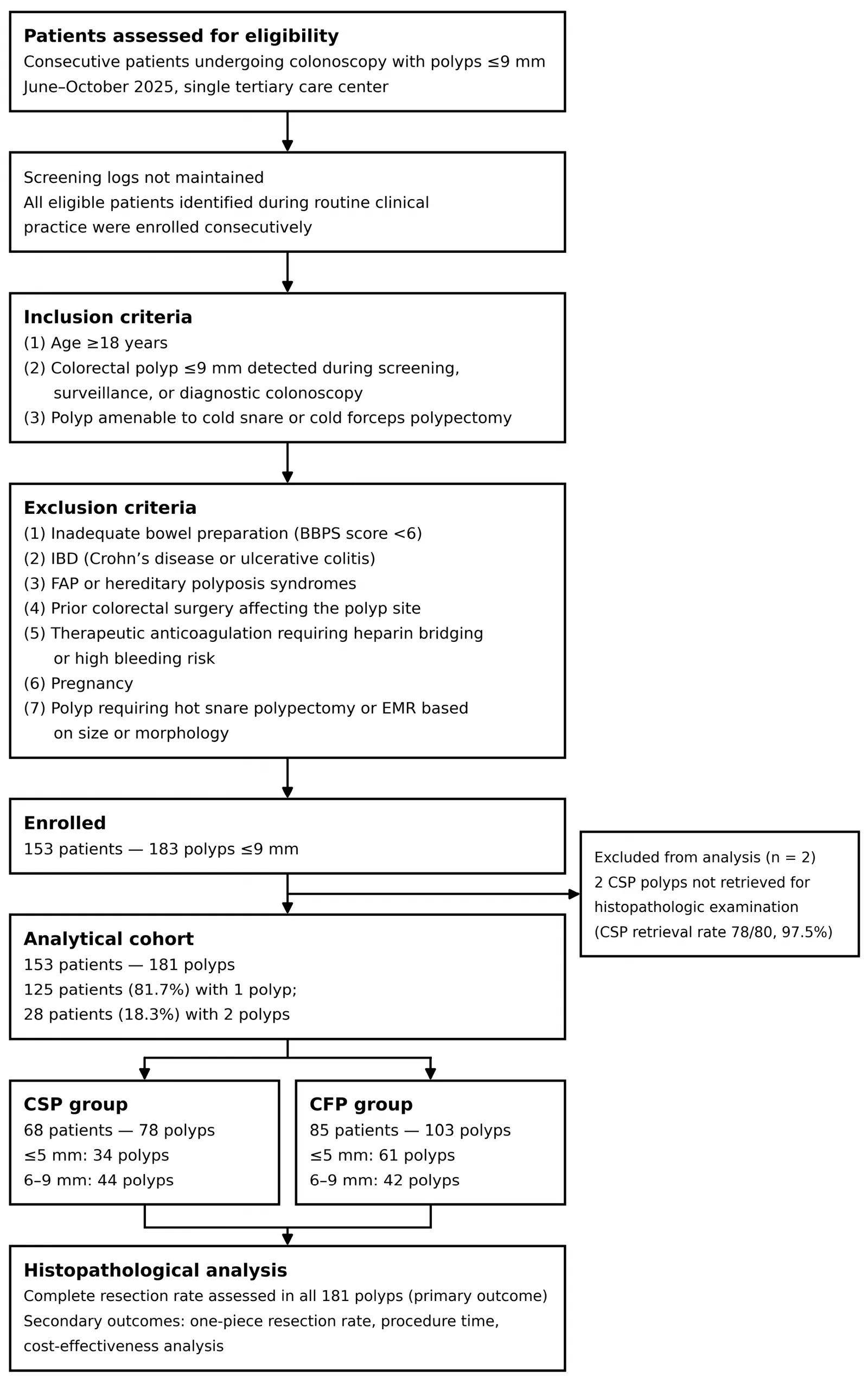
Flow diagram of patient and polyp selection. Consecutive patients undergoing colonoscopy with colorectal polyps ≤ 9 mm were assessed for eligibility between June and October 2025 at a single tertiary care center. After applying inclusion and exclusion criteria, 153 patients with 183 polyps were enrolled. Two polyps in the CSP group could not be retrieved for histopathologic examination and were excluded (CSP retrieval rate 78/80, 97.5%; CFP retrieval rate 103/103, 100%; overall 181/183, 98.9%), yielding an analytical cohort of 153 patients with 181 polyps allocated to CSP (68 patients, 78 polyps) or CFP (85 patients, 103 polyps) based on endoscopist preference and polyp characteristics. CSP: cold snare polypectomy; CFP: cold forceps polypectomy; BBPS: Boston Bowel Preparation Scale; IBD: inflammatory bowel disease; FAP: familial adenomatous polyposis; EMR: endoscopic mucosal resection.

During the 5-month study period (June–October 2025), consecutive patients undergoing colonoscopy with polyps ≤ 9 mm were assessed for eligibility. After applying inclusion and exclusion criteria, 153 patients with 183 polyps were enrolled (Figure 1). Two polyps in the CSP group could not be retrieved for histopathologic examination after polypectomy and were excluded from the analytical cohort (CSP retrieval rate: 78/80, 97.5%; CFP retrieval rate: 103/103, 100%; overall retrieval rate: 181/183, 98.9%). The final analytical cohort thus included 153 patients with 181 polyps. In the analytical cohort, 125 (81.7%) had one polyp and 28 (18.3%) had two polyps.

The CSP group included 68 patients with 78 polyps, while the CFP group included 85 patients with 103 polyps (Table 1). A large baseline imbalance was observed in the number of polyps per patient (SMD = +0.386, *p* = 0.022), with a higher proportion of single-polyp patients in the CSP group (89.7% vs. 75.3%). Other patient characteristics—including age, body mass index, sex, ASA class, Boston Bowel Preparation Scale score, and Charlson Comorbidity Index—showed no large imbalances between groups (all |SMD| ≤ 0.25; Table 1).

Polyp characteristics are presented in Table 2. Large baseline imbalances (|SMD| > 0.25) were observed for several polyp characteristics. The CSP group had larger polyps than the CFP group (median 6 mm (IQR, 5–7) vs. 5 mm (IQR, 4–6), SMD = +0.538, *p* < 0.001), with proportionally more polyps in the 6–9 mm size range (56.4% vs. 40.8%, SMD = +0.317, *p* = 0.037). Polyp location also showed a large imbalance, with distal colon location more frequent in the CSP group (61.5% vs. 47.6%, SMD = +0.283, *p* = 0.062). Paris classification subtypes showed smaller, sub-threshold imbalances (Table 2). Histopathologic type and advanced adenoma status were balanced between groups (all |SMD| < 0.10; Table 2). These large baseline imbalances in polyp size and location motivated the propensity score matched sensitivity analysis (described below) to address potential confounding from non-randomized technique selection.

### 3.2. Resection and Procedural Outcomes

Complete resection was achieved in 76 of 78 polyps (97.4%) in the CSP group versus 97 of 103 polyps (94.2%) in the CFP group (odds ratio (OR) = 2.35, 95% confidence interval (CI), 0.46–11.98; *p* = 0.29) (Table 4). This difference was not statistically significant, and the wide confidence interval reflects the small number of incomplete resection events (*n* = 8); the study was not powered to detect a difference in complete resection.

CSP demonstrated significantly higher one-piece resection rates compared to CFP (75/78 (96.2%) vs. 87/103 (84.5%); *p* = 0.011). Conversely, piecemeal resection occurred less frequently with CSP (3/78 (3.8%) vs. 16/103 (15.5%); OR = 0.22, 95% CI, 0.06–0.78), representing a 78% reduction in the odds of piecemeal resection with cold snare technique. Procedure time was notably shorter for CSP (median 3.2 min (IQR, 2.7–4.1) vs. 5.1 min (IQR, 4.3–7.2); *p* < 0.001). Accounting for intra-patient clustering with cluster-robust standard errors, and restricting the analysis to one randomly selected polyp per patient, did not materially change these estimates (Tables S2 and S3).

### 3.3. Safety Outcomes

A total of two procedure-related complications were observed during the study period, with comparable per-patient rates between groups (1/68 (1.5%) in the CSP group and 1/85 (1.2%) in the CFP group; OR = 1.25, 95% CI 0.08–20.42; *p* = 1.000) (Table 4). No perforations or post-polypectomy syndrome occurred in either group. In the CSP group, one intraprocedural bleeding event occurred during resection of a 9 mm tubular adenoma located in the rectum; bleeding was successfully managed with endoscopic hemoclip placement during the index procedure, with immediate hemostasis achieved. In the CFP group, one delayed bleeding event occurred on the first post-procedural day following resection of an 8 mm tubular adenoma located in the descending colon, which was managed conservatively without endoscopic intervention. Neither patient required hospitalization or red blood cell transfusion, and both events resolved without long-term sequelae.

### 3.4. Subgroup Analysis by Polyp Size

In exploratory subgroup analyses stratified by polyp size (Table 5), for diminutive polyps (≤5 mm), both complete resection (100% vs. 95.1%, *p* = 0.55) and piecemeal resection (0.0% vs. 6.6%, *p* = 0.29) rates were comparable between CSP and CFP. For small polyps (6–9 mm), complete resection rates were also comparable (95.5% vs. 92.9%, *p* = 0.673). However, CSP demonstrated a substantially lower piecemeal resection rate (3/44 (6.8%) vs. 12/42 (28.6%), *p* = 0.010), a 75% relative reduction. Given the modest subgroup sample sizes, these findings should be interpreted as hypothesis-generating.

### 3.5. Cost Analysis

**Figure 2:**
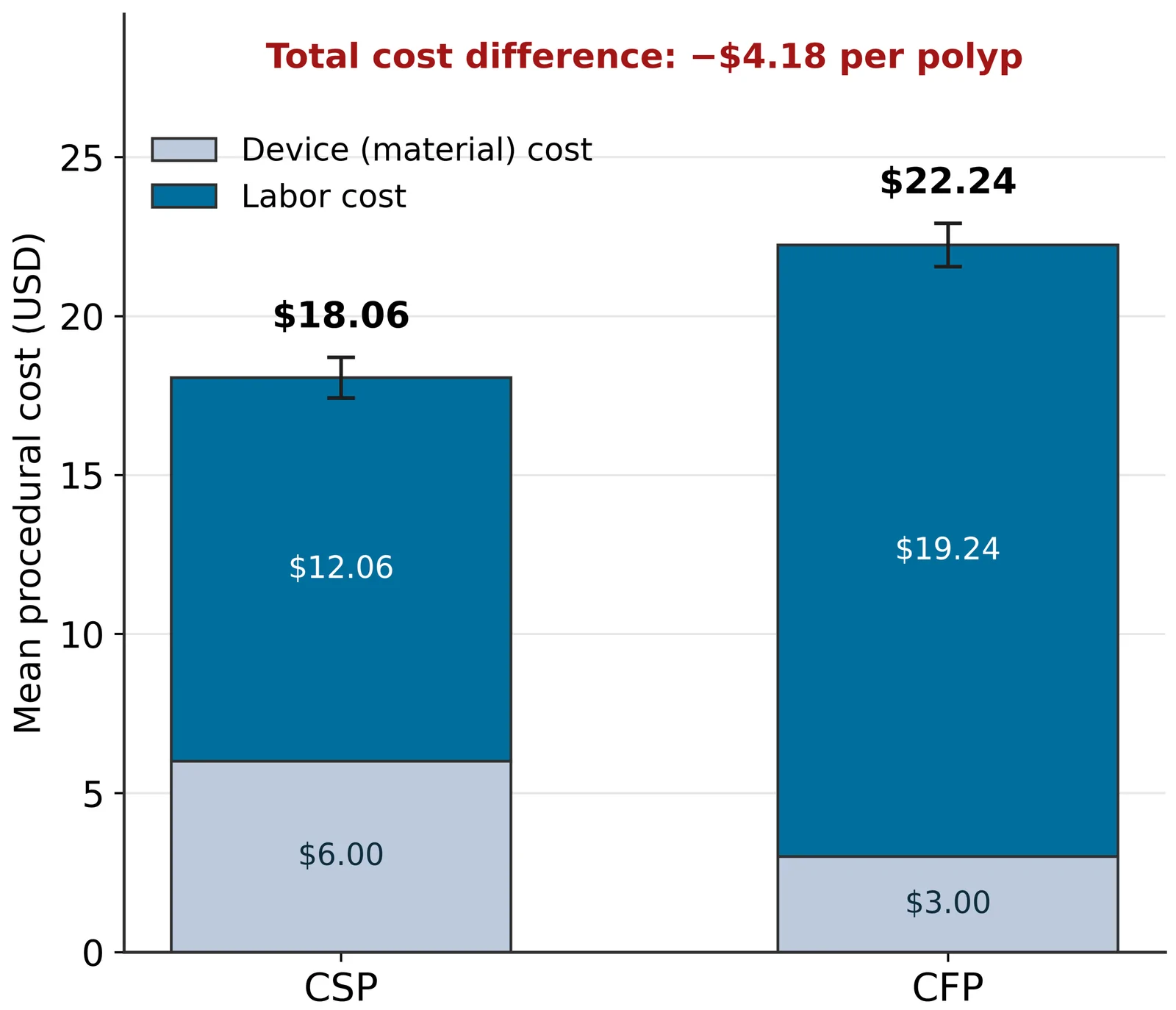
Decomposition of mean procedural cost per polyp by technique. Stacked bars show the mean procedural cost per polyp for cold snare polypectomy (CSP) and cold forceps polypectomy (CFP), separated into device (material) cost and labor cost. Although the device cost was higher for CSP ($6.00 vs. $3.00), its shorter procedure time yielded a lower labor cost ($12.06 vs. $19.24) and, consequently, a lower direct device-plus-labor cost ($18.06 vs. $22.24), corresponding to a mean direct device-plus-labor cost difference of −$4.18 per polyp in favor of CSP. Error bars represent the standard error of the mean direct device-plus-labor cost. CSP: cold snare polypectomy; CFP: cold forceps polypectomy.

Despite higher material costs for CSP ($6.00 vs. $3.00), the technique demonstrated lower labor costs ($12.06 ± 5.66 vs. $19.24 ± 6.94, *p* < 0.001) due to shorter procedure time (Table 6). Direct device-plus-labor cost was lower for CSP ($18.06 ± 5.66 vs. $22.24 ± 6.94, *p* < 0.001), representing a mean cost savings of $4.18 per polyp (Figure 2).

**Figure 3:**
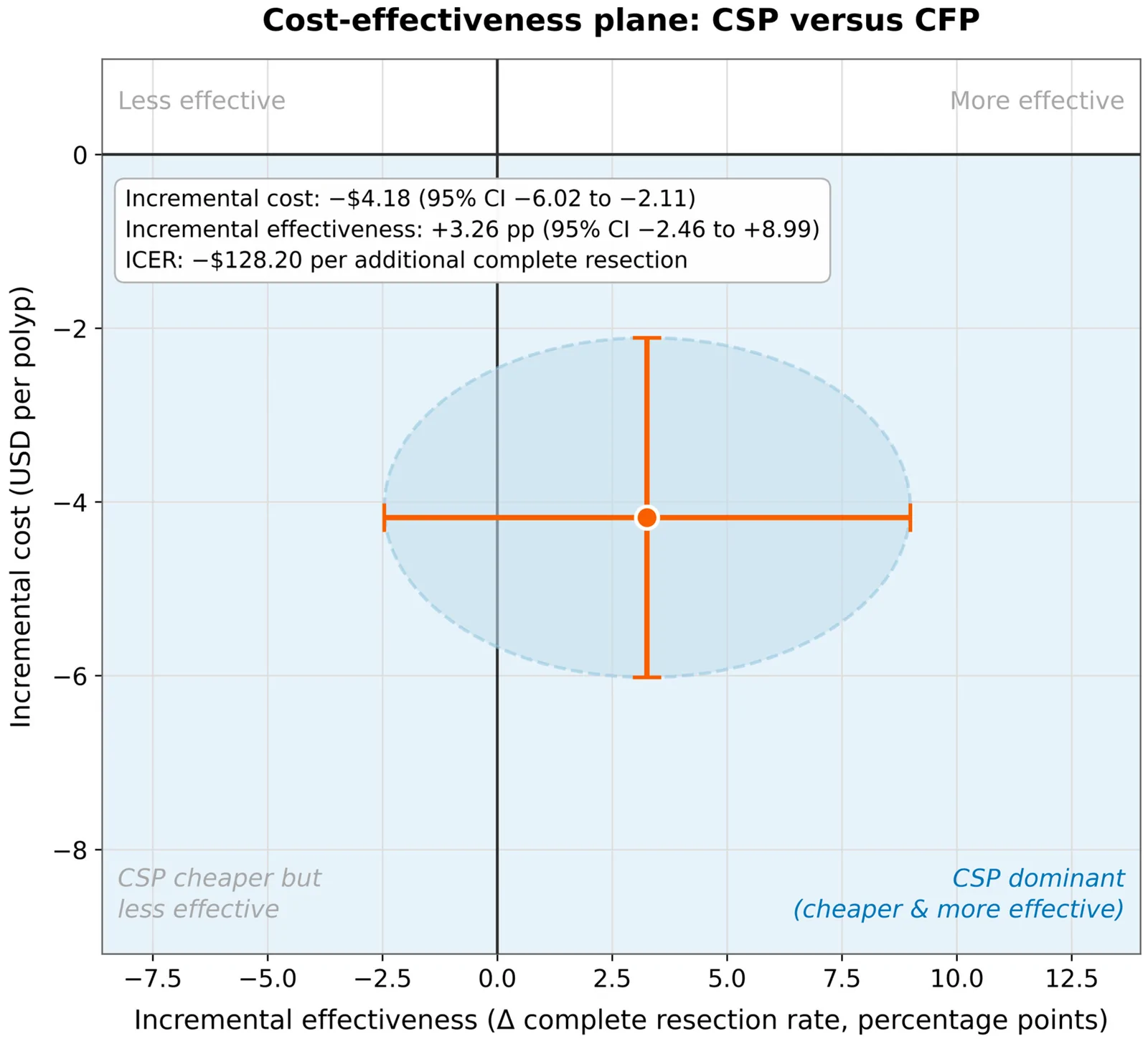
Cost-effectiveness plane comparing cold snare polypectomy (CSP) with cold forceps polypectomy (CFP) for small colorectal polyps. The orange point denotes the base-case estimate of incremental cost (−$4.18 per polyp) and incremental effectiveness (+3.26 percentage points in the complete resection rate) for CSP relative to CFP; orange bars indicate the corresponding 95% bootstrap confidence intervals (cost, −6.02–−2.11; effectiveness, −2.46–+8.99), and the dashed ellipse represents the joint 95% confidence region. As the incremental cost lies entirely below the horizontal axis while the effectiveness interval crosses the vertical axis, the estimate reflects cost-minimization at comparable effectiveness rather than statistical dominance. The incremental cost-effectiveness ratio (ICER) was −$128.20 per additional complete resection. Confidence intervals were estimated by bootstrap resampling with 10,000 iterations. CSP: cold snare polypectomy; CFP: cold forceps polypectomy; ICER: incremental cost-effectiveness ratio.

Cost per complete resection was lower for CSP ($18.54 vs. $23.61, *p* < 0.001), corresponding to $5.08 per successfully resected polyp. Because the between-technique difference in complete resection was not statistically significant, this comparison was interpreted within a cost-minimization framework: CSP had significantly lower costs (incremental cost: −$4.18; 95% CI, −6.02–−2.11) at a numerically higher but non-significant complete resection rate (+3.26 percentage points; 95% CI, −2.46–8.99). The incremental cost-effectiveness ratio (−$128.20; 95% CI, −410.50–−32.70 per additional complete resection) is reported for completeness; given the non-significant effectiveness difference, it reflects cost minimization at comparable effectiveness rather than cost-effectiveness or dominance (Figure 3).

Sensitivity analysis demonstrated that CSP remained cost-saving across all tested labor rates. The mean direct device-plus-labor cost difference was −$1.78 per polyp at $2 per minute (95% CI, −3.20–−0.41; *p* = 0.01)**,** −$4.18 at the base rate of $3 per minute (as reported above), and −$6.57 at $4 per minute (95% CI, −9.15–−3.69; *p* < 0.001), confirming that the cost advantage of CSP was robust across the range of institutional labor-cost assumptions tested.

### 3.6. Regression Analysis

In Firth penalized logistic regression accounting for the low number of incomplete resection events (*n* = 8, 4.4%; approximately 2 events per covariate), high-grade dysplasia emerged as the only variable independently associated with incomplete resection (OR = 7.07, 95% CI, 1.50–30.17; *p* = 0.016) (Table 7). The polypectomy technique showed a directionally favorable association favoring CSP (OR = 0.42, 95% CI, 0.07–1.77; *p* = 0.246) but did not reach statistical significance. Polyp size and location were not independent predictors (both *p* > 0.30; Table 7). The overall model was statistically significant (likelihood-ratio χ^2^ = 10.02, df = 4; *p* = 0.040). Given the small number of events and the resulting wide confidence intervals, these regression estimates should be interpreted as exploratory and hypothesis-generating.

### 3.7. Propensity Score Matched Analysis

From the initial cohort of 78 CSP and 103 CFP polyps, one-to-one nearest-neighbor matching produced 61 matched pairs (122 polyps in total; matching rates: 78.2% for CSP, 59.2% for CFP), with 17 CSP polyps (21.8%) and 42 CFP polyps (40.8%) remaining unmatched. All eight matching covariates achieved acceptable balance by the |SMD| < 0.25 criterion (Table S8), with continuous polyp size—the principal matching covariate—showing SMD = −0.071. The categorical ≤5 mm and 6–9 mm size subgroups showed SMDs of +0.266 and −0.266, respectively; these reflect categorical subgroup distributions on a derived dichotomization rather than imbalance on the underlying continuous matching covariate. High-grade dysplasia, which was not used in matching, showed a residual descriptive SMD of 0.160.

In the matched cohort, complete resection was achieved in 59 of 61 polyps (96.7%) in the CSP group versus 56 of 61 polyps (91.8%) in the CFP group (risk difference (RD) = +4.9%, 95% CI −3.3–+13.1; McNemar exact *p* = 0.453) (Table 3). The discordant pair distribution favored CSP (5 versus 2 pairs). Secondary outcomes were directionally consistent with the full-cohort analysis. CSP achieved a substantially higher one-piece resection rate (60/61 (98.4%) vs. 48/61 (78.7%); RD = +19.7%, 95% CI +9.8–+29.5; *p* < 0.001) and a correspondingly lower piecemeal resection rate (1/61 (1.6%) vs. 13/61 (21.3%); RD = −19.7%; *p* < 0.001). Procedure time was shorter with CSP (median 3.3 min (IQR 2.7–4.4) vs. 5.4 min (IQR 4.6–7.9); *p* < 0.001), and direct device-plus-labor cost was lower for CSP ($17.94 vs. $21.81; mean difference −$3.87; *p* < 0.001). The resulting ICER of −$78.70 (95% CI −294.30–+241.73) per additional complete resection indicates that CSP was cost-saving in the matched comparison, while the effectiveness gain did not reach statistical significance.

An exploratory subgroup analysis was restricted to the 24 matched pairs in which both members had polyps measuring 6–9 mm. Complete resection rates were similar (23/24 (95.8%) vs. 22/24 (91.7%); RD = +4.2%; *p* = 1.000). However, CSP demonstrated substantially higher one-piece resection (23/24 (95.8%) vs. 15/24 (62.5%); RD = +33.3%; *p* = 0.008) and a correspondingly lower piecemeal rate (1/24 (4.2%) vs. 9/24 (37.5%); RD = −33.3%; *p* = 0.008). Given the small sample size, these subgroup findings should be interpreted as hypothesis-generating.

### 3.8. Sensitivity Analysis in Neoplastic Polyps

In the sensitivity analysis restricted to neoplastic polyps (*n* = 134; 58 in the CSP group and 76 in the CFP group), the direction and magnitude of all primary and secondary outcomes were consistent with the full-cohort findings. Complete resection rates were 56/58 (96.6%) for CSP and 71/76 (93.4%) for CFP (risk difference +3.1%, 95% CI −4.2–+10.4; *p* = 0.698). The advantage of CSP in one-piece resection was preserved (96.6% vs. 82.9%; risk difference +13.7%, 95% CI +4.0–+23.3; *p* = 0.013), with a corresponding reduction in piecemeal resection (3.4% vs. 17.1%; risk difference −13.7%; *p* = 0.013). Procedure time remained shorter for CSP (median 3.30 min (IQR 2.70–4.28) vs. 5.85 (4.50–8.57); *p* < 0.001), and direct device-plus-labor cost was lower for CSP ($17.44 vs. $22.63; mean difference −$5.18, 95% CI −7.13–−3.21; *p* < 0.001). The cost per complete resection was $18.06 for CSP and $24.22 for CFP, yielding an ICER of −$165.62 (95% CI −1010.85–+954.44), showing cost-saving with a non-significant effectiveness gain, consistent with the matched cohort analysis. Detailed results are presented in Table S7.

## 4. Discussion

This prospective cohort study confirms the established clinical advantages of cold snare polypectomy over cold forceps polypectomy and provides novel cost-minimization evidence using real institutional pricing. Our clinical findings—significantly higher one-piece resection rates and shorter procedure times—are consistent with the existing randomized trial evidence underlying current guideline recommendations [7,8]. The novel contribution lies in our economic analysis, demonstrating that CSP is cost-saving in real-world institutional practice while achieving comparable resection completeness, providing supporting evidence for guideline-concordant practice and resource allocation.

These findings align with recent meta-analyses showing CSP’s advantages over CFP for diminutive and small polyps. Kamal et al. reported in a 2023 meta-analysis of 9 randomized controlled trials that complete resection rates were markedly higher with CSP compared to standard-capacity CFP (OR 1.68, 95% CI 1.09–2.58) [20]. The TINYPOLYP trial demonstrated that CFP was noninferior to CSP for very small polyps (≤3 mm) [21]. Taken together, these findings suggest that the benefit of CSP becomes more pronounced as polyp size increases beyond the ≤3 mm threshold examined in TINYPOLYP, particularly in the 6–9 mm range, consistent with our subgroup analysis showing a 75% reduction in piecemeal resection with CSP for 6–9 mm polyps.

Although randomized trials and meta-analyses have compared CSP and CFP, these have focused on clinical outcomes such as complete resection, tissue retrieval, and procedure time rather than economic endpoints [20,22]. Cold forceps polypectomy nonetheless remains a frequently used technique for small polyps in routine practice [22], valued for its simplicity and low per-device cost, despite guideline preference for CSP. Existing economic evaluations of cold snare polypectomy have compared it with endoscopic mucosal resection rather than cold forceps [23]. Our study addresses this gap by providing prospective cost-minimization evidence with transparent device and labor cost accounting. Device costs were obtained from institutional purchasing contracts, while labor costs were derived from institutional payroll and overhead accounting, yielding an approximate blended rate of $3 per minute, within the range of published microcosting studies ($0.68–4.31 per minute) [24]. Sensitivity analysis across labor rates of $2–4 per minute confirmed CSP’s cost advantage was robust across different labor cost assumptions. Despite higher device costs for CSP ($6.00 vs. $3.00), the technique resulted in lower direct device-plus-labor costs ($18.06 vs. $22.24, *p* < 0.001) due to reduced procedure time. The mean cost savings of $4.18 per polyp represents a 19% cost reduction. This cost advantage is primarily driven by shorter procedure times (median 3.2 vs. 5.1 min), which reduces labor costs and increases endoscopy unit throughput.

In our cost-minimization analysis, CSP achieved significantly lower costs while delivering procedural and resection outcomes consistent with established evidence. The ICER of −$128.20 per additional complete resection indicates that CSP is cost-saving at comparable complete resection rates; as the effectiveness difference was not statistically significant, this represents a cost-minimization rather than a cost-effectiveness trade-off, in which comparable effectiveness is assumed rather than statistically confirmed. This finding has important implications for healthcare systems seeking to optimize colonoscopy screening programs without compromising quality. The time efficiency of CSP aligns with published literature. Kim et al., in their 2023 multicenter randomized trial comparing CSP with cold endoscopic mucosal resection, reported that CSP reduced procedure time (45.8 vs. 87.6 s, *p* < 0.001) while maintaining equivalent complete resection rates [25]. Similarly, the large Taiwanese TACOS trial demonstrated that CSP reduced mean polypectomy time by 44 s compared to hot snare polypectomy (119.0 vs. 162.9 s) [26]. Notably, in TINYPOLYP (≤3 mm), CSP required longer procedure time than CFP (42.3 vs. 23.2 s, *p* < 0.001), reflecting the single-bite efficiency of forceps for very small lesions. This size-dependent crossover supports our finding that CSP’s time advantage emerges for larger polyps where multiple forceps bites become necessary [21]. This time efficiency not only reduces costs but also minimizes patient discomfort and allows for higher throughput in busy endoscopy units.

Our exploratory subgroup analysis revealed that the advantage of CSP was most pronounced for polyps in the 6–9 mm size range, where piecemeal resection occurred in only 6.8% of CSP cases compared to 28.6% of CFP cases. This difference is clinically important because incomplete resection is a recognized risk factor for interval colorectal cancer [5]. One-piece resection ensures complete histopathologic evaluation and reduces the risk of residual neoplastic tissue, which may contribute to adenoma recurrence and interval cancers. Recent meta-analyses have demonstrated that CSP can achieve high complete resection rates for polyps up to 10 mm in size [27]. Emerging evidence also supports its efficacy even for larger polyps when appropriate technique is employed [28]. The 2024 updated ESGE guidelines recommend CSP as the preferred technique for small (6–9 mm) non-pedunculated colorectal polyps and recommend against cold forceps polypectomy because of its high rate of incomplete resection [8]—a recommendation our subgroup data reinforce, with a fourfold higher piecemeal resection rate for CFP in this size range. The same guideline emphasizes resource-efficient polypectomy as integral to effective colorectal cancer screening, a priority to which our economic findings add direct comparative evidence.

In Firth penalized multivariable analysis—chosen given the low event rate (*n* = 8; ~2 events per covariate)—high-grade dysplasia was the only variable independently associated with incomplete resection (OR = 7.07, 95% CI 1.50–30.17; *p* = 0.016). CSP showed a directionally favorable association (OR = 0.42, 95% CI 0.07–1.77) that did not reach significance, likely reflecting limited statistical power; these penalized-regression estimates are therefore exploratory and hypothesis-generating. This should be interpreted alongside the descriptive advantage of CSP in one-piece resection (96.2% vs. 84.5%; *p* = 0.011), the 75% reduction of piecemeal resection for 6–9 mm polyps (6.8% vs. 28.6%; *p* = 0.010), and the numerically higher histopathologic complete resection rate (97.4% vs. 94.2%). The HGD association likely reflects the biological behavior of more advanced lesions and the technical challenge of resecting dysplastic tissue [29].

To address potential confounding from non-randomized technique selection—manifested as large baseline imbalances in polyp size and location—we performed propensity score matched analysis as a sensitivity analysis. In the matched cohort of 61 pairs balanced on eight matching covariates (polyp size, multiple lesion status, location, Boston Bowel Preparation Scale score, and Paris classification categories), the substantial one-piece resection advantage of CSP was preserved and the effect estimate was strengthened (98.4% vs. 78.7% in the matched cohort vs. 96.2% vs. 84.5% in the full cohort; paired OR = 25.00, 95% CI 1.48–422.26 (Haldane–Anscombe corrected); *p* < 0.001), with all twelve discordant pairs favoring CSP (Table 3). Complete resection rates remained directionally consistent with the full-cohort analysis (96.7% vs. 91.8%; paired OR = 2.50; McNemar exact *p* = 0.453), though the difference did not reach statistical significance in either cohort, likely reflecting the low overall event rate of incomplete resection. The matched-cohort ICER of −$78.70 per additional complete resection (95% CI −294.30–+241.73) indicated that CSP remained cost-saving in the matched comparison, although the wider confidence interval crossing zero reflects the smaller matched sample size. In an exploratory analysis restricted to 24 matched pairs in which both members had 6–9 mm polyps, CSP showed a pronounced one-piece resection advantage (95.8% vs. 62.5%; RD = +33.3%; McNemar *p* = 0.008; paired OR = 17.00, 95% CI 0.98–294.55 (Haldane–Anscombe corrected), with a corresponding 33-percentage-point reduction in piecemeal resection; the wide confidence interval reflects the small number of discordant pairs, and this exploratory subgroup warrants cautious interpretation. This subgroup signal supports the current ESGE 2024 guideline recommendation of CSP for the removal of 6–9 mm colorectal polyps [8]. The mechanistic explanation, consistent with the ESGE-recommended technique of including a 1–2 mm margin of normal tissue around the polyp [8,22], is that the snare’s continuous tissue grip and en bloc capture of this margin become increasingly important relative to serial forceps bites as lesion diameter approaches the upper bound of the cold-resection range. To quantify robustness to unmeasured confounding, E-values were calculated on the risk-ratio scale [16]. The E-value was 7.54 for piecemeal resection, indicating that this association would be robust to substantial residual confounding, whereas the more modest E-values for one-piece resection (1.54) and complete resection (1.22, with a confidence-interval limit of 1.00 consistent with the non-significant primary endpoint) indicate that these associations could be sensitive to moderate unmeasured confounding and warrant appropriate caution. Although direct CSP-versus-CFP propensity matched comparisons remain scarce, the use of matched designs in colorectal polypectomy research [30] complements the evidence from randomized trials in the absence of head-to-head randomization, and our findings extend the existing randomized-trial meta-analytic evidence base [22] by providing confounding-adjusted estimates that converge with the full-cohort and Firth regression analyses. This matching improves internal validity but cannot eliminate residual confounding inherent to operator-selected treatment strategies; the convergent estimates therefore strengthen the internal consistency of the findings rather than establishing causal superiority.

A further sensitivity analysis restricted to neoplastic polyps (*n* = 134), for which the primary outcome definition is most directly applicable, yielded results directionally consistent with both the full-cohort and propensity score-matched analyses [31]. Complete resection rates remained numerically higher with CSP (96.6% vs. 93.4%; OR = 1.97; *p* = 0.698), though the difference did not reach statistical significance—consistent with the sparse-event pattern (only 7 incomplete resection events in this neoplastic-restricted analysis) similarly observed in the full cohort and matched analyses, which limits both statistical power and the precision of effect estimates [32]. The one-piece resection advantage of CSP was preserved (96.6% vs. 82.9%; OR = 5.78, 95% CI 1.25–26.73; *p* = 0.013), with a corresponding reduction in piecemeal resection (3.4% vs. 17.1%; *p* = 0.013). Procedure time remained shorter with CSP (3.30 vs. 5.85 min; *p* < 0.001), and the cost advantage was maintained (mean difference −$5.18; *p* < 0.001; ICER −$165.62 per additional complete resection, indicating cost-savings) (Table S7). The consistent direction of all primary and secondary outcomes across the full-cohort, propensity score-matched, and neoplastic-restricted analyses indicates that the observed CSP advantages are unlikely to be primarily artifacts of non-neoplastic lesion inclusion.

No perforations or post-polypectomy syndrome occurred in either group, and bleeding events were infrequent and minor (one intraprocedural bleeding in the CSP group managed endoscopically with hemoclip placement, and one delayed bleeding on the first post-procedural day in the CFP group managed conservatively); both events resolved without hospitalization, transfusion, or long-term sequelae. These findings are consistent with multiple large series reporting low complication rates with cold techniques [26,33]. The avoidance of electrocautery eliminates the risk of thermal injury, post-polypectomy electrocoagulation syndrome, and delayed bleeding associated with eschar separation. Combined with its established efficacy in the literature and our cost-minimization findings, these results support CSP as a guideline-recommended approach for small colorectal polyps.

Several limitations of this study warrant consideration. First, the absence of systematic screening logs prevents precise calculation of enrollment rate, detailed exclusion breakdown, and formal verification of strict consecutive enrollment, although all eligible patients identified during routine clinical practice were included. Second, this was a single-center study at a tertiary care teaching hospital, which may limit generalizability to community settings; however, our endoscopists’ experience level (~1000 colonoscopies annually each) is representative of practicing endoscopists. External validation in independent, multicenter cohorts with diverse practice settings and cost structures would be a valuable next step in confirming the broader applicability of these findings. Third, the study was not blinded. Endoscopist awareness of the polypectomy technique at the time of base biopsy collection is unavoidable given that the techniques are visually distinct. More importantly, pathologists were not blinded to the resection technique during histopathologic evaluation, which may introduce detection bias affecting the primary outcome of complete resection—although objective outcomes such as procedure time and cost calculations are less susceptible. A sensitivity analysis reclassifying a plausible range of complete resections as incomplete indicated that the primary comparison remained non-significant under both non-differential and technique-differential misclassification assumptions (Table S5). Future studies should consider blinded central pathologic review to address this limitation. Fourth, complete resection was assessed by base biopsy rather than post-polypectomy site biopsy at a later colonoscopy, which is considered the gold standard; base biopsy thus represents a surrogate endpoint and, although an accepted outcome measure in polypectomy studies, does not by itself establish durable histologic eradication. Moreover, because the base biopsies sampled only the center of the resection base, this approach may not reliably exclude residual tissue at the lateral margins of the post-polypectomy defect, particularly after piecemeal cold forceps resection. Fifth, we did not include long-term follow-up to assess adenoma recurrence rates, which would provide definitive evidence of technique effectiveness. Sixth, the sample size may have been insufficient to detect statistically meaningful differences in complete resection rates, despite a numerically higher completeness rate (97.4% vs. 94.2%). Detecting small absolute differences when both techniques achieve high baseline rates (>94%) requires substantially larger sample sizes; larger multicenter studies are warranted to formally confirm the higher completeness observed for CSP at the descriptive level. Seventh, several constraints apply to the economic analysis. Costs were based on institutional pricing at a single center and expressed in U.S. dollars using a study-period average exchange rate; because cost structures and currency values vary across healthcare systems and over time, the absolute cost estimates may not generalize, although the time-efficiency advantage of CSP that drives them is likely consistent across settings. The advantage would be expected to hold in any setting where the labor rate exceeds an estimated break-even value of approximately U.S. $1.25 per minute—well below our institutional rate—below which the higher device cost of CSP would no longer be offset by its shorter procedure time. The analysis was conducted from a provider perspective and confined to direct device and procedural labor costs; sedation, pathology, complication management, repeat procedures, and downstream costs were not captured, and no health-utility (e.g., quality-adjusted life-year) valuation or discounting was applied. Accordingly, the findings represent a short-horizon cost-consequence comparison rather than a full cost-utility evaluation. Eighth, technique selection was at the endoscopist’s discretion based on polyp characteristics rather than randomized, which could introduce selection bias. Although Firth penalized multivariable analysis adjusted for polyp size, location, and high-grade dysplasia, residual confounding from unmeasured variables—such as detailed Paris morphology sub-types, endoscopist identity and technique-specific experience, withdrawal time, adenoma detection rate, learning-curve effects, lesion accessibility, and the endoscopist’s histologic suspicion, lesion-appearance assessment, and perceived technical difficulty—cannot be entirely excluded (quantified by the E-value analysis; Table S6); moreover, Paris classification was assigned prospectively by the performing endoscopist without independent verification, and interobserver agreement was not assessed. Ninth, our propensity score matching analysis had specific methodological limitations: 59 of the 181 polyps could not be matched within the caliper, a residual descriptive imbalance persisted for high-grade dysplasia (SMD = 0.160; not used in matching, as histopathology was determined after polypectomy), and matching was performed at the polyp rather than patient level, which does not fully account for within-patient clustering of multiple lesions. Tenth, the neoplastic-restricted sensitivity analysis (*n* = 134) was conducted post-hoc and reduced the analytical sample compared to the full cohort, with wider confidence intervals for binary outcomes. Finally, polyp retrieval was unsuccessful in 2 of 80 CSP polyps (2.5%); these were excluded from the analytical cohort as histopathologic confirmation was not feasible. The CSP retrieval rate (97.5%) is consistent with published rates for cold snare polypectomy (typically 95–100%). Even under a conservative classification of these 2 polyps as incomplete resections, the CSP complete resection rate would be 95.0% (76/80), remaining directionally consistent with and numerically higher than the CFP rate (94.2%) (Table S4). This indicates that exclusion of non-retrieved polyps did not materially affect the primary outcome interpretation.

## 5. Conclusions

This prospective cohort study demonstrates that cold snare polypectomy achieves higher one-piece resection rates and shorter procedure times at lower direct device-plus-labor cost than cold forceps polypectomy for small colorectal polyps, with no statistically significant difference in complete resection or observed adverse events. These findings were consistent across Firth-penalized multivariable, propensity score-matched, and neoplastic-restricted sensitivity analyses. Beyond confirming established clinical advantages, this study provides novel cost-minimization evidence using real institutional pricing: within this cost-minimization framework, CSP is cost-saving while also improving one-piece resection outcomes, supporting its adoption as a guideline-recommended technique for small colorectal polyp removal. In this cohort of polyps ≤ 9 mm, the procedural time advantage of CSP emerged particularly for larger (6–9 mm) lesions, where multiple forceps bites become necessary—a size-dependent efficiency pattern that is clinically relevant for technique selection. As healthcare systems increasingly focus on value-based care, CSP offers a practical approach to improving both quality and efficiency in colorectal cancer prevention.

## Figures and Tables

**Table 1:** Baseline characteristics of patients.

Variable	Total (*n* = 153)	CSP (*n* = 68)	CFP (*n* = 85)	SMD	*p*
Age (years), median (IQR)	61 (51–69)	62 (52–69)	61 (51–68)	+0.071	0.636
BMI (kg/m^2^), median (IQR)	27.9 (24.8–30.2)	28.3 (25.2–31.0)	27.7 (24.5–30.0)	+0.145	0.386
BBPS, median (IQR)	7 (7–8)	7 (7–8)	7 (7–8)	+0.101	0.324
CCI, median (IQR)	2 (1–4)	3 (1.8–4)	2 (1–4)	+0.087	0.602
Male sex	75 (49.0%)	36 (52.9%)	39 (45.9%)	+0.142	0.385
ASA I	22 (14.4%)	12 (17.6%)	10 (11.8%)	+0.167	0.718
ASA II	88 (57.5%)	38 (55.9%)	50 (58.8%)	−0.059	
ASA III	35 (22.9%)	14 (20.6%)	21 (24.7%)	−0.098	
ASA IV	8 (5.2%)	4 (5.9%)	4 (4.7%)	+0.053	
1 polyp per patient	125 (81.7%)	61 (89.7%)	64 (75.3%)	+0.386	0.022 ^a^
2 polyps per patient	28 (18.3%)	7 (10.3%)	21 (24.7%)	−0.386	

^a^ *p* < 0.05. SMD: Standardized mean difference; |SMD| > 0.10 indicates meaningful imbalance and |SMD| > 0.25 indicates large imbalance. For continuous variables, SMDs were calculated from means and standard deviations even when medians and IQRs are reported. ASA categories are shown with per-category SMDs. BMI: Body mass index; BBPS: Boston Bowel Preparation Scale; CCI: Charlson Comorbidity Index; ASA: American Society of Anesthesiologists classification; CSP: Cold snare polypectomy; CFP: Cold forceps polypectomy; IQR: Interquartile range.

**Table 2:** Polyp characteristics.

Variable	Total (*n* = 181)	CSP (*n* = 78)	CFP (*n* = 103)	SMD	*p*
Polyp size (mm), median (IQR)	5 (4–7)	6 (5–7)	5 (4–6)	+0.538	<0.001 ^b^
≤5 mm	95 (52.5%)	34 (43.6%)	61 (59.2%)	−0.317	0.037 ^a^
6–9 mm	86 (47.5%)	44 (56.4%)	42 (40.8%)	+0.317	
Proximal colon	84 (46.4%)	30 (38.5%)	54 (52.4%)	−0.283	0.062
Distal colon	97 (53.6%)	48 (61.5%)	49 (47.6%)	+0.283	
Non-neoplastic	47 (26.0%)	20 (25.6%)	27 (26.2%)	−0.013	0.941
Low-grade dysplasia	117 (64.6%)	50 (64.1%)	67 (65.0%)	−0.020	
High-grade dysplasia	17 (9.4%)	8 (10.3%)	9 (8.7%)	+0.052	
Advanced adenoma	30 (16.6%)	14 (17.9%)	16 (15.5%)	+0.065	0.910
Paris classification, *n* (%)					
0-Is	109 (60.2%)	47 (60.3%)	62 (60.2%)	+0.001	0.640
0-IIa	46 (25.4%)	17 (21.8%)	29 (28.2%)	−0.147	
0-Ip	11 (6.1%)	5 (6.4%)	6 (5.8%)	+0.024	
0-Isp	7 (3.9%)	4 (5.1%)	3 (2.9%)	+0.113	
0-IIb	8 (4.4%)	5 (6.4%)	3 (2.9%)	+0.166	

^a^ *p* < 0.05, ^b^ *p* < 0.001. Continuous variables compared using Mann-Whitney U test; categorical variables using chi-square test. *p* values for multi-category variables (dysplasia, Paris classification) represent overall comparison across categories. SMD: Standardized mean difference; |SMD| > 0.10 indicates meaningful imbalance and |SMD| > 0.25 indicates large imbalance. For continuous variables, SMDs were calculated from means and standard deviations even when medians and IQRs are reported. Per-category SMDs are reported for multi-level variables (histopathology, Paris classification). CSP: Cold snare polypectomy; CFP: Cold forceps polypectomy; IQR: Interquartile range.

**Table 3:** Primary and secondary outcomes in the propensity score matched cohort.

Outcome	CSP (*n* = 61)	CFP (*n* = 61)	Effect/Difference	*p* Value
***Primary outcome***				
Complete resection, *n*/N (%)	59/61 (96.7)	56/61 (91.8)	OR = 2.50 (95% CI 0.49–12.89); RD = +4.9% (95% CI −3.3–+13.1)	0.453 ^a^
***Secondary outcomes***				
One-piece resection, *n*/N (%)	60/61 (98.4)	48/61 (78.7)	OR = 25.00d (95% CI 1.48–422.26); RD = +19.7% (95% CI +9.8–+29.5)	<0.001 ^a,c^
Piecemeal resection, *n*/N (%)	1/61 (1.6)	13/61 (21.3)	RD = −19.7%	<0.001 ^a,c^
Procedure time (min), median [IQR]	3.3 (2.7–4.4)	5.4 (4.6–7.9)	−2.1 min	<0.001 ^b,c^
Direct device-plus-labor cost (USD), mean ± SD	17.94 ± 5.52	21.81 ± 6.79	−3.87 USD	<0.001 ^b,c^
***Subgroup analysis: 6–9 mm polyps (n = 24 matched pairs)***				
Complete resection, *n*/N (%)	23/24 (95.8)	22/24 (91.7)	OR = 2.00 (95% CI 0.18–22.06); RD = +4.2%	1.000 ^a^
One-piece resection, *n*/N (%)	23/24 (95.8)	15/24 (62.5)	OR = 17.00 ^d^ (95% CI 0.98–294.55); RD = +33.3%	0.008 ^a,c^
Piecemeal resection, *n*/N (%)	1/24 (4.2)	9/24 (37.5)	RD = −33.3%	0.008 ^a,c^
***Cost analysis in matched cohort***				
Incremental cost, USD	−3.87	(95% CI −5.85–−1.89)	-	-
Incremental effectiveness	+4.9 percentage points	(95% CI −3.3–+13.1)	-	-
ICER (USD per additional complete resection)	−78.70	(95% CI −294.30–+241.73)	-	-

^a^ McNemar’s exact test for paired binary outcomes. ^b^ Wilcoxon signed-rank test for paired continuous outcomes. ^c^ *p* < 0.05. ^d^ Haldane-Anscombe continuity correction (+0.5 added to each cell) applied due to zero CFP-only discordant pairs. Odds ratios (OR) for binary outcomes represent paired (McNemar) ORs in the matched cohort, with 95% CIs estimated using the Wald log-OR method. Discordant pair counts: complete resection in matched cohort = 5 (CSP-favoring) and 2 (CFP-favoring); one-piece resection = 12 and 0; complete resection in 6–9 mm subgroup = 2 and 1; one-piece in 6–9 mm subgroup = 8 and 0. The 6–9 mm subgroup is defined as matched pairs in which both the CSP and CFP polyp were 6–9 mm (*n* = 24 pairs); this analysis is exploratory given the small sample size. Risk difference (RD) calculated as CSP minus CFP; 95% CIs estimated by bootstrap with 10,000 iterations at the matched-pair level. CSP: Cold snare polypectomy; CFP: Cold forceps polypectomy; OR: Odds ratio; RD: Risk difference; IQR: Interquartile range; SD: Standard deviation; CI: Confidence interval; USD: United States dollars; ICER: Incremental cost-effectiveness ratio.

**Table 4:** Clinical outcomes.

Outcome	CSP	CFP	Effect Size	95% CI	*p*
**Primary outcome**					
Complete resection, *n*/N (%)	76/78 (97.4)	97/103 (94.2)	OR = 2.35; RD = 3.3%	OR 0.46–11.98; RD −3.8–9.8	0.290
**Secondary outcomes**					
Piecemeal resection, *n*/N (%)	3/78 (3.8)	16/103 (15.5)	OR = 0.22; RD = −11.7%	OR 0.06–0.78; RD −20.3–−2.7	0.011 ^a^
Procedure time (min), median (IQR)	3.2 (2.7–4.1)	5.1 (4.3–7.2)	−1.9 min	-	<0.001 ^b^
Any complication (per patient), *n*/N (%)	1/68 (1.5)	1/85 (1.2)	OR = 1.25; RD = 0.3%	OR 0.08–20.42; RD −5.0–6.8	1.000

^a^ *p* < 0.05, ^b^ *p* < 0.001. OR for piecemeal resection represents odds of piecemeal resection with CSP vs. CFP; OR < 1 indicates lower odds with CSP. OR: Odds ratio; RD: Risk difference; CI: Confidence interval; IQR: Interquartile range; CSP: Cold snare polypectomy; CFP: Cold forceps polypectomy.

**Table 5:** Outcomes stratified by polyp size.

Variable	CSP ≤ 5 mm	CFP ≤ 5 mm	*p*	CSP 6–9 mm	CFP 6–9 mm	*p*
Proximal colon, *n* (%)	14/34 (41.2)	29/61 (47.5)	0.668	16/44 (36.4)	25/42 (59.5)	0.051
Non-neoplastic, *n* (%)	8/34 (23.5)	16/61 (26.2)	0.811	12/44 (27.3)	11/42 (26.2)	1.000
Low-grade dysplasia, *n* (%)	22/34 (64.7)	40/61 (65.6)	1.000	28/44 (63.6)	27/42 (64.3)	1.000
High-grade dysplasia, *n* (%)	4/34 (11.8)	5/61 (8.2)	0.717	4/44 (9.1)	4/42 (9.5)	1.000
Advanced adenoma, *n* (%)	5/34 (14.7)	9/61 (14.8)	1.000	9/44 (20.5)	7/42 (16.7)	0.784
Complete resection, *n*/N (%)	34/34 (100.0)	58/61 (95.1)	0.550	42/44 (95.5)	39/42 (92.9)	0.673
Piecemeal resection, *n*/N (%)	0/34 (0.0)	4/61 (6.6)	0.293	3/44 (6.8)	12/42 (28.6)	0.010 ^a^

^a^ *p* < 0.05. CSP: Cold snare polypectomy; CFP: Cold forceps polypectomy.

**Table 6:** Cost analysis and incremental cost outcomes.

Component	CSP	CFP	Effect/Difference	95% CI	*p*
**A. Cost analysis**					
Material cost (USD)	6.00 ± 0.00	3.00 ± 0.00	+3.00 USD	N/A	N/A
Labor cost (USD)	12.06 ± 5.66	19.24 ± 6.94	−7.18 USD	-	<0.001 ^b^
Direct device-plus-labor cost (USD)	18.06 ± 5.66	22.24 ± 6.94	−4.18 USD	−6.02–−2.11	<0.001 ^b^
Complete resection rate	76/78 (97.4%)	97/103 (94.2%)	+3.26%	−2.46–8.99	0.290
Cost per complete resection (USD)	18.54	23.61	−5.08 USD	−7.60–−2.56	<0.001 ^b^
**B. Incremental cost analysis**					
	**CSP vs. CFP**				
Incremental cost	−4.18 USD		-	−6.02–−2.11	-
Incremental effectiveness	+3.26 percentage points		-	−2.46–8.99	-
ICER	−128.20 USD per additional complete resection		-	−410.50–−32.70	-

^b^ *p* < 0.001. Bootstrap confidence intervals estimated using 10,000 iterations at the patient level. A negative ICER indicates that CSP is cost-saving at comparable complete resection rates. CSP: Cold snare polypectomy; CFP: Cold forceps polypectomy; CI: Confidence interval; USD: United States dollars; ICER: Incremental cost-effectiveness ratio. Values in Table 6B represent incremental differences (CSP minus CFP) rather than group-specific values.

**Table 7:** Firth penalized logistic regression for predictors of incomplete resection (*n* = 181 polyps, 8 events).

Variable	OR	95% CI	*p* Value
Technique (CSP vs. CFP)	0.42	0.07–1.77	0.246
Polyp size (6–9 mm vs. ≤5 mm)	2.02	0.51–9.19	0.316
Location (proximal vs. distal)	1.09	0.26–4.59	0.900
High-grade dysplasia (present vs. absent)	7.07	1.50–30.17	0.016

CSP: cold snare polypectomy; CFP: cold forceps polypectomy; OR: odds ratio; CI: confidence interval. Outcome: histopathologically confirmed incomplete resection (8/181, 4.4%). Overall model: likelihood-ratio χ^2^ = 10.02, df = 4, *p* = 0.040.

## Data Availability

The data presented in this study are available on request from the corresponding author due to privacy restrictions regarding patient information.

## Supplementary Materials

The following supporting information can be downloaded at: https://www.mdpi.com/article/10.3390/healthcare14162656/s1, Table S1: Normality testing (Shapiro–Wilk) of cost and procedure-time variables by technique; Table S2: Sensitivity of the binary outcomes to intra-patient clustering: unadjusted versus patient-clustered odds ratios; Table S3: Sensitivity analysis restricted to one randomly selected polyp per patient; Table S4: Worst-case (intention-to-treat) sensitivity analysis for complete resection; Table S5: Sensitivity of the primary outcome (complete resection) to potential outcome misclassification; Table S6: E-value analysis for potential unmeasured confounding; Table S7: Sensitivity analysis–outcomes restricted to neoplastic polyps; Table S8: Baseline characteristics in the propensity score matched cohort; Table S9: Univariable analysis for incomplete resection; Table S10: Comparison of CSP and CFP by dysplasia grade; Table S11: Comparison by advanced adenoma status; Figure S1: Covariate balance before and after propensity score matching (Love plot).
