# Supplementary material for: Cost-Minimization and Procedural Outcomes of Cold Snare Versus Cold Forceps Polypectomy for Small Colorectal Polyps: A Prospective Cohort Study Using Real Institutional Pricing
Source: Healthcare (Basel). 2026 Aug 21;14(16):2656. doi: 10.3390/healthcare14162656 (PMC13512676; doi:10.3390/healthcare14162656)
Supplement: Supplementary file 1 [file healthcare-14-02656-s001.zip › healthcare-4452623-supplementary.pdf]

Table S1. Normality testing (Shapiro–Wilk) of cost and procedure-time variables by technique.

Variable	CSP: W (P)	CFP: W (P)	Distribution
Material cost	constant (N/A)	constant (N/A)	No within-group variance
Labor cost	0.86 (<0.001)	0.91 (<0.001)	Non-normal
Direct device-plus-labor cost	0.86 (<0.001)	0.91 (<0.001)	Non-normal
Procedure time	0.86 (<0.001)	0.91 (<0.001)	Non-normal

Shapiro–Wilk test. Material (device) cost was fixed within each technique (CSP USD 6.00; CFP USD 3.00) and therefore had no within-group variance, so a normality test was not applicable. Labor cost, direct device-plus-labor cost, and procedure time were non-normally distributed in both groups, supporting the use of non-parametric tests (Mann–Whitney U) for these variables. Procedure time is reported as median [IQR]; cost variables are summarized as mean ± SD in the main text to reflect their budgetary interpretation, while significance was assessed non-parametrically. W: Shapiro–Wilk statistic; N/A: not applicable.

Table S2. Sensitivity of the binary outcomes to intra-patient clustering: unadjusted versus patient-clustered odds ratios.

Outcome	Unadjusted OR [95% CI]	Patient-clustered OR [95% CI]
Complete resection	2.35 [0.46–11.98]	2.35 [0.46–12.09]
One-piece resection	4.60 [1.29–16.39]	4.60 [1.28–16.46]
Piecemeal resection	0.22 [0.06–0.78]	0.22 [0.06–0.78]

Odds ratios (OR) compare CSP versus CFP (reference) and reproduce the primary polyp-level analysis reported in the main text and Table 4. Patient-clustered 95% confidence intervals (CI) were obtained using cluster-robust (Huber–White sandwich) standard errors with the patient as the clustering unit. The point estimates were identical and the confidence intervals essentially unchanged, so statistical significance was unaltered for every outcome: complete resection remained non-significant (CI includes 1), whereas one-piece and piecemeal resection remained significant (CI excludes 1). This confirms that intra-patient clustering did not materially affect inference. CSP: cold snare polypectomy; CFP: cold forceps polypectomy; OR: odds ratio; CI: confidence interval.

Table S3. Sensitivity analysis restricted to one randomly selected polyp per patient (n = 153).

Outcome	CSP	CFP	P
Complete resection	97.2%	92.7%	0.286
One-piece resection	95.8%	80.5%	0.006
Piecemeal resection	4.2%	19.5%	0.006
Procedure time, median (min)	3.3	5.1	<0.001
Direct device-plus-labor cost, median (USD)	15.9	18.3	<0.001

One polyp per patient was randomly selected (n = 153; CSP 71, CFP 82; random seed fixed for reproducibility), yielding a data set with strictly independent observations. Binary outcomes were compared using the Fisher exact test and continuous outcomes using the Mann–Whitney U test. Results were consistent with the full polyp-level analysis, further supporting robustness to intra-patient clustering. Rates differ marginally from the full-cohort values because this is a distinct single-polyp-per-patient subsample. CSP: cold snare polypectomy; CFP: cold forceps polypectomy.

Table S4. Worst-case (intention-to-treat) sensitivity analysis for complete resection.

Scenario	CSP	CFP	P
Base-case (analytical cohort)	76/78 (97.4%)	97/103 (94.2%)	0.29
Worst-case (2 unretrieved CSP polyps classified as incomplete)	76/80 (95.0%)	97/103 (94.2%)	0.81

Intention-to-treat worst-case analysis reclassifying the two CSP polyps not retrieved for histopathological examination as incomplete resections (CSP denominator increased from 78 to 80). The chi-square test was used, consistent with the primary analysis; the base-case P value reproduces that reported for complete resection in Table 4. Complete resection remained numerically higher than, or comparable to, CFP and statistically non-significant under both scenarios, indicating that exclusion of the unretrieved polyps did not materially bias the primary endpoint. CSP: cold snare polypectomy; CFP: cold forceps polypectomy.

Table S5. Sensitivity of the primary outcome (complete resection) to potential outcome misclassification.

Scenario	CSP	CFP	Difference (pp)	P
Base-case (observed)	76/78 (97.4%)	97/103 (94.2%)	+3.3	0.290
Non-differential, 5%	72/78 (92.3%)	92/103 (89.3%)	+3.0	0.495
Non-differential, 10%	68/78 (87.2%)	87/103 (84.5%)	+2.7	0.606
Differential, 5% (CSP only)	72/78 (92.3%)	97/103 (94.2%)	−1.9	0.617
Differential, 10% (CSP only)	68/78 (87.2%)	97/103 (94.2%)	−7.0	0.101

Because pathologists were not blinded to the polypectomy technique, the primary outcome may be susceptible to detection bias. The between-technique comparison was re-evaluated under a plausible range of outcome misclassification. In the non-differential scenarios, an equal proportion of complete resections in both groups was reclassified as incomplete; in the more conservative differential scenarios, only CSP complete resections were reclassified, corresponding to the direction of bias that would arise if awareness of the technique favored CSP. The difference remained non-significant across all scenarios. Under the extreme differential assumption, statistical significance was reached only when at least 13% of CSP complete resections were reclassified, and in that case the difference favored CFP rather than CSP. These findings indicate that the primary comparison—and the cost-minimization interpretation based on comparable resection completeness—is robust to plausible outcome misclassification. Comparisons used the chi-square test, consistent with the primary analysis. pp: percentage points; CSP: cold snare polypectomy; CFP: cold forceps polypectomy.

Table S6. E-value analysis for potential unmeasured confounding.

Outcome	RR (CSP vs CFP) [95% CI]	E-value (point estimate)	E-value (CI limit)
One-piece resection	1.14 [1.04–1.25]	1.54	1.23
Piecemeal resection	0.25 [0.08–0.82]	7.54	1.74
Complete resection	1.03 [0.97–1.10]	1.22	1.00

The E-value is the minimum strength of association, on the risk-ratio scale, that an unmeasured confounder would need to have with both the technique and the outcome to fully explain away the observed association, over and above the measured covariates. Risk ratios were computed directly from the observed proportions reported in the main text. An E-value of 1.00 for complete resection reflects a confidence interval that already includes the null, consistent with the non-significant primary endpoint. While the E-value for piecemeal resection was large (7.54), indicating robustness to substantial residual confounding, the more modest E-values for one-piece and complete resection indicate that these

associations could be sensitive to moderate residual confounding and should be interpreted with appropriate caution. RR: risk ratio; CI: confidence interval.

Table S7. Sensitivity analysis: outcomes restricted to neoplastic polyps (n = 134; CSP = 58, CFP = 76)

Outcome	CSP (n = 58)	CFP (n = 76)	Effect / Difference (95% CI)	P value
Primary outcome				
Complete resection, n/N (%)	56/58 (96.6)	71/76 (93.4)	OR = 1.97 (0.37–10.55); RD = +3.1% (−4.2 to +10.4)	0.698a
Secondary outcomes				
One-piece resection, n/N (%)	56/58 (96.6)	63/76 (82.9)	OR = 5.78 (1.25–26.73); RD = +13.7% (+4.0 to +23.3)	0.013a,c
Piecemeal resection, n/N (%)	2/58 (3.4)	13/76 (17.1)	OR = 0.17 (0.04–0.80); RD = −13.7% (−23.3 to −4.0)	0.013a,c
Procedure time (min), median [IQR]	3.30 [2.70–4.28]	5.85 [4.50–8.57]	−2.55 min	<0.001b,c
Direct device-plus-labor cost (USD), mean ± SD	17.44 ± 4.83	22.63 ± 6.94	Mean diff = −5.18 (−7.13 to −3.21)	<0.001b,c
Cost analysis				
Incremental cost (USD)	−5.18	(95% CI −7.13 to −3.21)	—	—
Incremental effectiveness	+3.1 pp	(95% CI −4.2 to +10.4)	—	—
Cost per complete resection (USD)	18.06	24.22	Savings = −6.16	—
ICER (USD per additional complete resection)	−165.62	(95% CI −1,010.85 to +954.44)	Cost-saving	—

aFisher's exact test for binary outcomes. bMann–Whitney U test for continuous outcomes. cP < 0.05. Odds ratios (OR) for binary outcomes were calculated using the Wald method with 95% confidence intervals; Haldane–Anscombe continuity correction (+0.5 added to each cell) was applied when any cell contained zero counts. Risk differences (RD) were calculated as CSP minus CFP; 95% CIs for proportions estimated by the Wald method, and 95% CIs for mean cost difference and ICER estimated by bootstrap resampling with 10,000 iterations. A negative incremental cost combined with a non-negative point estimate for incremental effectiveness indicates that CSP is cost-saving in the neoplastic subset**.** CSP: Cold snare polypectomy; CFP: Cold forceps polypectomy; OR: Odds ratio; RD: Risk difference; CI: Confidence interval; IQR: Interquartile range; SD: Standard deviation; USD: United States dollars; pp: Percentage points; ICER: Incremental cost-effectiveness ratio.

Table S8. Baseline characteristics in the propensity score matched cohort (n = 122 polyps; 61 matched pairs)

Variable	CSP (n=61)	CFP (n=61)	SMD
Demographic characteristics			
Age (years), median [IQR]	62 [52–69]	59 [51–67]	0.142
Male sex, n (%)	30 (49.2)	27 (44.3)	0.099
BMI (kg/m^2), median [IQR]	28.3 [25.1–31.2]	27.3 [24.5–29.8]	0.143
Matching covariates (used in propensity score model)			
Polyp size (mm), median [IQR]	5 [4–7]	6 [5–7]	−0.071
≤5 mm, n (%)	31 (50.8)	23 (37.7)	+0.266
6–9 mm, n (%)	30 (49.2)	38 (62.3)	−0.266
Multiple lesion, n (%)	14 (23.0)	16 (26.2)	−0.076
Proximal colon, n (%)	30 (49.2)	30 (49.2)	0.000
BBPS, median [IQR]	7 [7–8]	7 [7–8]	−0.016
Paris classification, n (%)			
0-Is	35 (57.4)	38 (62.3)	−0.100
0-IIa	14 (23.0)	14 (23.0)	0.000
0-Ip	5 (8.2)	5 (8.2)	0.000
0-Isp	3 (4.9)	2 (3.3)	0.083
0-IIb	4 (6.6)	2 (3.3)	0.152
Histopathology, n (%)			
Tubular adenoma	41 (67.2)	40 (65.6)	0.035
Hyperplastic polyp	12 (19.7)	16 (26.2)	−0.156
Tubulovillous adenoma	5 (8.2)	4 (6.6)	0.063
Sessile serrated lesion	3 (4.9)	1 (1.6)	0.185
High-grade dysplasia, n (%)	8 (13.1)	5 (8.2)	0.160

Standardized mean differences (SMD) are reported; |SMD| < 0.25 indicates acceptable balance. Matching covariates are those used in the propensity score model. Polyp size was included as a continuous variable in the propensity model (SMD = −0.071); the dichotomized ≤5 mm and 6–9 mm SMDs reflect categorical subgroup distributions rather than imbalance on the underlying continuous matching covariate. Demographic characteristics (age, sex, BMI) and histopathology are presented for descriptive purposes only and were not used in the matching procedure. CSP: Cold snare polypectomy; CFP: Cold forceps

polypectomy; SMD: Standardized mean difference; BMI: Body mass index; IQR: Interquartile range; BBPS: Boston Bowel Preparation Scale.

Table S9. Univariable analysis for incomplete resection

Variable	OR	Robust 95% CI	P
Technique (CSP vs CFP)	0.43	0.08–2.19	0.306
Polyp size (mm)	1.66	1.00–2.76	0.052
Size group (6–9 mm vs ≤5 mm)	1.89	0.44–8.15	0.392
Proximal location (vs distal)	0.68	0.16–2.99	0.611
Piecemeal resection (vs one piece)	1.23	0.14–10.67	0.851
Low-grade dysplasia (vs non-neoplastic)	0.53	0.13–2.21	0.384
High-grade dysplasia (vs non-neoplastic)	6.81	1.47–31.58	0.014[a]
Advanced adenoma	3.24	0.71–14.79	0.128
Age (years)	0.99	0.92–1.07	0.847
BMI (kg/m^2)	0.95	0.82–1.09	0.480
BBPS	1.30	0.50–3.39	0.591
CCI	1.23	0.75–2.00	0.418
Male sex	1.81	0.41–7.89	0.431
Two polyps per patient	0.31	0.04–2.54	0.273

aP < 0.05. Each variable tested separately. OR: Odds ratio; CI: Confidence interval; CSP: Cold snare polypectomy; CFP: Cold forceps polypectomy; BMI: Body mass index; BBPS: Boston Bowel Preparation Scale; CCI: Charlson Comorbidity Index.

Table S10. Comparison of CSP and CFP by dysplasia grade

Variable	CSP Non-neoplastic	CFP Non-neoplastic	P	CSP LGD	CFP LGD	P	CSP HGD	CFP HGD	P
Polyp size ≤5 mm, n (%)	8/20 (40.0)	16/27 (59.3)	0.244	22/50 (44.0)	40/67 (59.7)	0.134	4/8 (50.0)	5/9 (55.6)	1.000
Polyp size 6–9 mm, n (%)	12/20 (60.0)	11/27 (40.7)	0.244	28/50 (56.0)	27/67 (40.3)	0.134	4/8 (50.0)	4/9 (44.4)	1.000
Proximal colon, n (%)	10/20 (50.0)	14/27 (51.9)	1.000	17/50 (34.0)	36/67 (53.7)	0.040[a]	3/8 (37.5)	4/9 (44.4)	1.000

Variable	CSP Non-neoplastic	CFP Non-neoplastic	P	CSP LGD	CFP LGD	P	CSP HGD	CFP HGD	P
Advanced adenoma, n (%)	0/20 (0.0)	0/27 (0.0)	1.000	6/50 (12.0)	7/67 (10.4)	1.000	8/8 (100.0)	9/9 (100.0)	1.000

aP < 0.05. CSP: Cold snare polypectomy; CFP: Cold forceps polypectomy; LGD: Low-grade dysplasia; HGD: High-grade dysplasia.

Table S11. Comparison by advanced adenoma status

Variable	CSP Non-adenomatous	CFP Non-adenomatous	P	CSP Non-advanced	CFP Non-advanced	P	CSP Advanced	CFP Advanced	P
Proximal colon, n (%)	10/20 (50.0)	14/27 (51.9)	1.000	16/44 (36.4)	32/60 (53.3)	0.112	4/14 (28.6)	8/16 (50.0)	0.284
Non-neoplastic, n (%)	20/20 (100.0)	27/27 (100.0)	1.000	0/44 (0.0)	0/60 (0.0)	1.000	0/14 (0.0)	0/16 (0.0)	1.000
Low-grade dysplasia, n (%)	0/20 (0.0)	0/27 (0.0)	1.000	44/44 (100.0)	60/60 (100.0)	1.000	6/14 (42.9)	7/16 (43.8)	1.000
High-grade dysplasia, n (%)	0/20 (0.0)	0/27 (0.0)	1.000	0/44 (0.0)	0/60 (0.0)	1.000	8/14 (57.1)	9/16 (56.2)	1.000

CSP: Cold snare polypectomy; CFP: Cold forceps polypectomy.

Figure S1. Covariate balance before and after propensity score matching (Love plot).

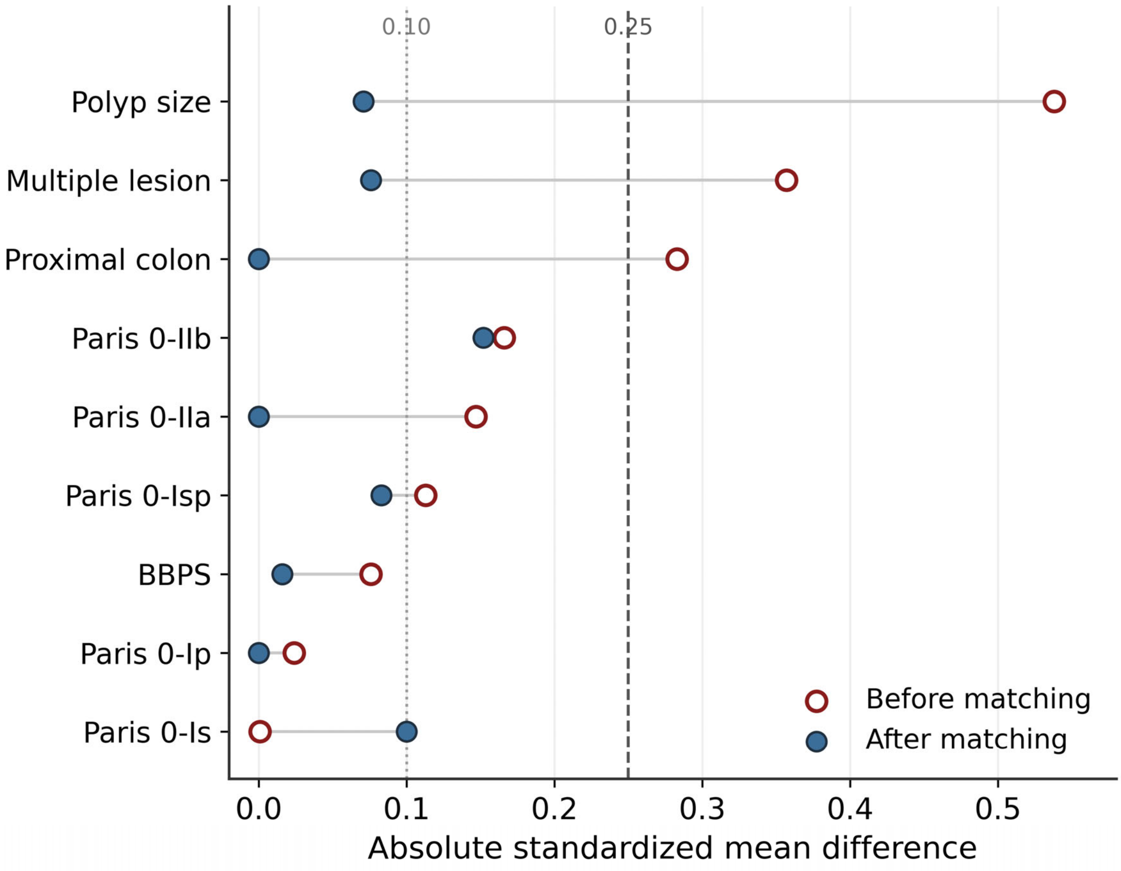

Absolute standardized mean differences (|SMD|) for the eight matching covariates (with Paris type 0-IIb shown as the reference category) are shown before matching (full cohort) and after one-to-one nearest-neighbor matching (matched cohort). Vertical reference lines denote the |SMD| thresholds of 0.10 and 0.25. After matching, all covariates fell below the 0.25 threshold, indicating acceptable balance. |SMD|: absolute standardized mean difference.
